# Turning the gaze: challenges of involving biomedical researchers in community engagement with research in Patan, Nepal

**DOI:** 10.1080/09581596.2018.1443203

**Published:** 2018-02-22

**Authors:** Siân Aggett

**Affiliations:** a Global Studies, University of Sussex, Brighton, UK

**Keywords:** Engagement, community, participation

## Abstract

Global health funding bodies are increasingly promoting and offering specific funding support for public and community engagement activities, in addition to research and programme funding. In the context of this growing commitment to engagement work, we need to find ways to better support contextually appropriate and meaningful exchanges between researchers and community members. I argue that, rather than focusing solely on how to involve communities in engagement with global health research, we should also pay attention to the quality and depth of the involvement of researchers themselves. This is an often overlooked dimension of community engagement in both practice and the literature. In this paper, I present three contextual factors, which created logistical and attitudinal obstacles for researchers’ involvement in meaningful engagement in a global health research unit in Nepal. These comprised implicit and explicit messages from funders, institutional and disciplinary hierarchies and educational experiences. Lessons were drawn from an exploration of the successes and failures of two participatory arts projects connected to the research unit in 2015 and 2016. Both projects intended to foster mutual understanding between researchers and members of their research population. As an engagement practitioner and ethnographic researcher, I documented the processes.

Enteric fever is a major public health problem in the Kathmandu valley, Nepal (Karkey et al., 2016). During preliminary conversations about a study designed to trial a water filter intervention, researchers from the Oxford University Clinical Research Programme in Nepal (OUCRU-NP), based at Patan Hospital, experienced resistance to participation in the study from residents within the area. So as to understand community concerns, two participatory arts projects – Sacred Water^1^ and Jeewan Jal^2^ – were initiated and were led by an artist from Vietnam and myself, respectively. The projects aimed to use conversations and collaborative art-making to generate opportunities for meaningful dialogue between researchers and community members, and to foster appreciation and understanding between the actors involved (Kester, 2004; Phillips, 2011). This aim was achieved with varying degrees of success. I show here, the projects followed the logic (and funder imperatives) of community engagement, but the researchers themselves were difficult to bring into the project activities.

Below, I describe these projects and explore the challenges encountered in involving medical research staff. I found three contextual factors that created obstacles for researcher involvement in engagement activities: implicit and explicit messages from funders that create ambiguity around the value of engagement, institutional and disciplinary hierarchies that influence task prioritisation, and educational experiences that undermine confidence in and the status of creative and dialogic activities. Findings indicate that establishing engagement activities in medical research settings uncritically and without adequate support may fail to create the intended two-way interactions (Research Councils UK [RCUK], 2010).

I argue that funders need to carefully consider what is required of both researchers and engagement practitioners in order to implement community engagement in global health research, and how they might more appropriately encourage and support genuinely dialogical forms of engagement.

## Defining public and community engagement

In his recent book on a history of global health, Packard (2016) identified that failure to engage local communities has been a central feature of most global health interventions since the 1930s. Funders of biomedical research, such as the Wellcome Trust and the Bill and Melinda Gates Foundation, are now increasingly offering support for public and community engagement in both locally and internationally funded programmes (Lavery et al., 2010). Still, the definition of engagement can be vague or unclear, and there is a lack of critical conversation regarding the aims and how to achieve them. By definition, ‘engagement’ differs from the public understanding of science in that it is ‘a two-way process, involving interaction and listening, with the goal of generating mutual benefit’ (National Coordinating Centre for Public Engagement, n.d.). Community engagement can be traced to development practices brought into global health research in response to a call to see stronger representation and participation of research communities, particularly vulnerable people (MacQueen, Bhan, Frohlich, Holzer, & Sugarman, 2015). Whilst public engagement attempts to engage with a broad and general group of non-specialists, community engagement focuses on a specific group associated with a research programme or project, identified through demographic criteria, geographic area or particular disease (Dunn, 2012; Hamlyn et al., 2015). However, the distinction is not always clear and often the terms are used interchangeably (Aggett, Dunn, & Vincent, 2012), as they are within this article, given that the projects outlined featured outputs (exhibitions) that intended to reach broad audiences, as well as a processes which aimed to foster dialogue and promote understanding between researchers and members of their research community.

It is worth noting that the emphasis within both terms is on engaging those outside of research, rather than engaging researchers. The literature tends to mirror this bias. This is despite concerns being raised that researcher involvement in engagement is not always as committed as hoped. Logistical pressures such as time, resource, reward and recognition, as well as demographic attributes such as age and gender, can influence the inclination of given researchers to engage (Hamlyn et al., 2015). This paper is a provocation to engagement practitioners and researchers to turn the gaze and enter this much-needed conversation.

I use the term ‘meaningful engagement’ in a considered way. For me, engagement is meaningful when people critically examine their own knowledge and express this without fear, to others who are receptive and responsive to this expression, bearing in mind that there will always be a muddying of the waters in the process of assimilation and interpretation (Falade & Coultas, 2017; Nichter, 2008). This concept of meaningful engagement is principally informed by scholars within international development, and specifically participatory development practice. Whilst engagement might not define itself by the underpinning principles and values purported to ground participatory development (Reason & Bradbury, 2008), parallels can be drawn between participatory development and a wider ethic that community engagement often ascribes to: ‘community empowerment’, ‘raising voice’ and promoting ‘equitable exchange and mutual understanding’. Therefore, criticism within international development of ‘tokenistic’ (Bell, 2004), ‘manipulative’ or ‘teleguided’ participatory activities (Rahnema, 2010) may also be pertinent to endeavours of engagement in global health. Rahnema (ibid.) indicated that spheres of power around development itself maybe fostering projects with little likelihood of challenging structural forces.

Engagement in global health is situated within wider fields of interest and power, which may or may not permit genuine exchange, community representation or empowerment. Whilst rhetoric promoting engagement in global health may emphasise dialogue, in my experience practice can neglect qualities contingent for dialogue. I believe that in order for engagement to be meaningful, we must pay attention to ensuring that what participants (researchers and community members alike) express is representative rather than tokenism or ventriloquism (Cornwall & Fujita, 2012). In her work on participatory video, Plush (2015) drew attention to the importance of representation alongside recognition of and responses to ‘voices raised’. Morrison and Dearden (2013) agree, asserting that in order to avoid tokenism, expressions need to be both ‘understandable and deemed valid by health professionals’.

## Study context and methods

Patan, also known as Lalitpur, is the second-largest city in the Kathmandu Valley. The Oxford University Clinical Research Unit-Nepal (OURCU-NP) is an infectious disease research unit based at the government-run Patan Hospital. The unit employs approximately 20 staff, all Nepali. They include microbiologists, clinicians, research nurses and community medical assistants (CMAs); the latter do the majority of community-based work, including patient recruitment, treatment delivery and data collection.

The population of Nepal is made up of multiple ethnic identity groups, with differing social and cultural practices. Newar is the most numerous ethnic identity group within the research area, however, increased immigration has brought increased diversification in the demographic of Patan (Gellner & Quigley, 1995). Most senior researchers, although from Kathmandu, were from outside of Patan and belonged to other ethnic/caste identities. This meant that they often had limited knowledge of the customs, relationships and beliefs within their research community compared to others within their research teams, such as the CMAs. With the increase in community-based epidemiological studies since the early 2000s, this has increased importance, and was a principle driver for the projects, which prompted this paper.

Other than some notable exceptions, there is little precedent for public or community engagement with global health research in Nepal.^3^ Before the participatory arts projects, Sacred Water and Jeewan Jal, OUCRU-NP’s engagement work had been limited to one-on-one interactions to address instrumental needs of research (patient recruitment, consent and data collection), rather than more prolonged interactions aimed at building understanding between researchers and their research community.

The internal drive for a more prolonged and interactive engagement at OUCRU-NP originated from experiences in the design stages of a 2013 community-based study, where Patan community members expressed dissatisfaction with a proposed randomised controlled trial (RCT) of bio-sand water filters. The lead researcher, Dr R, had not predicted a degree of intra-household cohesion, which made the planned randomisation of treatment difficult to justify and to implement. Recognising the need to better understand the research community, Dr R approached the engagement manager at the sister research unit in Vietnam (OUCRU-VN) for support. This led to two projects. The first, Sacred Water, was a participatory arts project conducted over the first half of 2015, led by a Vietnamese artist and funded through a Wellcome Trust International Engagement Award. I led a second participatory arts project, Jeewan Jal, from March to July 2016, which was funded through a Wellcome Trust ‘Ethics and Society’ award. The former project consisted of a series of arts workshops with female participants from local women’s groups. Whereas, Jeewan Jal, worked with an inter-caste and mixed-gender group of 11 young Nepali adults from various academic backgrounds. Both projects explored issues of water and health, and culminated in community exhibitions of artworks. Jeewan Jal also created and performed a community play entitled ‘Panika Gunjanharu: Echoes of Water’. Although the participating groups within these two projects differed, both attempted to draw medical research staff into ‘engaged’ interactions with participants throughout the processes. It is the success or failure of these attempts that are the focus of this paper.

I was invited to support Sacred Water logistically and to include it as a case study in my doctoral research (to explore the potential of participatory arts practice to create spaces for equitable dialogue and knowledge exchange between biomedics and research communities).^4^ Jeewan Jal forms a second doctoral case study. I hoped that the projects might deepen researchers’ understanding of the complexity of community, including the attitudes, behaviours and knowledge of research subjects (something global health is criticised for overlooking or overly simplifying (Nichter, 2008)).

At the request of the unit director, I conducted a community engagement training session with OUCRU-NP staff, including CMAs, research nurses, the director and other researchers. This took place before either project had begun, and used informal teaching methods eliciting existing interest and understandings of engagement. With attendees’ permission, this was sound-recorded, and contributed to the ethnographic data. At the end of the projects, I conducted 11 individual semi-structured interviews (Bryman, 2004) with staff from across levels of seniority. These interviews used thematic prompts developed from the open coding of field-notes (Strauss & Corbin, 1998), which documented informal conversations with staff about the challenges to their involvement in engagement. These included logistical considerations as well as attitudinal and more structural ones. I then transcribed and coded the interviews using NVivo. All data were collected with informants’ consent, and has been anonymised as far as possible. Key figures are Dr E, Dr L, Dr R and Dr Y. Other research participants are identified by their professional roles. The research was approved by the University of Sussex’s ethics review board.

My research was primarily ethnographic (Brewer, 2000), yet I also drew on participatory and informal learning methods (Chambers, 2002) to explore the relationships, exchanges and knowledge forms circulating within these community engagement processes. Methods drew upon participant observation and field journaling, paying attention to my own relationships, actions and emotions as well as those of others. It involved a lot of ‘hanging out’ and conversations over cups of tea with research staff in professional spaces of the office or laboratory, or at social events such as public talks and weddings. I was committed to being open, listening and approaching all interactions as a learner.

## Findings: challenges involving researchers in engagement activities

Numerous factors generated obstacles for researcher involvement in engagement processes. These fitted within the three overarching contextual factors: implicit and explicit messages from funders that create ambiguity around the value of engagement, institutional and disciplinary hierarchies that influence task prioritisation, and educational experiences that undermine confidence in and the status of creative and dialogic activities.

### Implicit and explicit messages from funding institutions

Above, I described the internal impetus for the engagement projects within OUCRU-NP, with the growing recognition that there was a need to better understand the research community. However, the processes were also driven by an external stimulus: efforts to promote engagement from other institutions within the global health network, particularly OUCRU-VN and the Wellcome Trust. The Wellcome Trust – like many other funding bodies – is increasingly encouraging community engagement, using rhetoric around promoting informed and inclusive conversation, dialogue and debate (Wellcome, 2017). Despite this message being implicit in funder behaviour, reporting structures and grant-giving provide clouded, if not contradictory, descriptions and explanations. A representative from the engagement team at Wellcome Trust visited Kathmandu during the Sacred Water project, demonstrating funder commitment. Conversely, there was little formal recognition for research staff involvement in engagement, or other incentive structures such as opportunities for accredited training. This was commented on in the group discussion in advance of the projects:

Discussion Prompt: I would like to do engagement but…?

Programme Director: There is a lack of funds, money, time, authority, approval. I need a trained team. (OUCRU-NP discussion exercise, January 2015)

The engagement manager in OUCRU-VN, and advisor to Sacred Water and Jeewan Jal, elaborated, stating that ‘Funders judge people by the number of publications they have … If it’s taking up people’s time … away from doing science that can generate a publication, then they won’t do it…’ There are questions within Wellcome’s research grant applications inviting applicants to describe engagement plans; however, the engagement manager confessed, most engagement written into these plans only involves researchers to a cursory degree.

Messages around engagement’s aims and values are not necessarily clear in other organisations, nor in the literature (Lavery, 2016). In a time-scarce work environment with little capacity or understanding of engagement, such activities understandably become secondary.

### Institutional hierarchies

When I asked whether community engagement was part of medical research work, the answer from all groups interviewed was invariably ‘Yes’. However, engagement clearly sat towards the bottom of a hierarchy of valuable work within the research institution. The quantifiable tasks of participant recruitment, data collection and publication were all valued over building understanding within the research community.

It was clear that CMAs felt their work was considered to be less important than the data analysis and publication work of more senior researchers. Dr R spoke of one CMA’s feelings after attending an engagement workshop in London: ‘He sort of realised that he did important work. That people actually saw his work’. This lack of institutional recognition of community facing work resulted in the people who were tasked with doing it feeling overworked and undervalued. This, in turn, resulted in reluctance to undertake additional engagement-related work. I mused over this with the director, who agreed that engagement was undervalued. As a remedy, he suggested that I try to ‘make it look exciting and fun…’

In response, I sought to create engagement activities that could be construed as fun. However, in doing so, I felt a tension. Whilst I argue the value of a playful space in which people and ideas can coincide and interact without conflict, the suggestion was that this should be the principle justification for research staff involvement in these activities. Although it was unintentional, I felt my work belittled, and that the challenges the projects originally hoped to address were at risk of being neglected. In addition, the use of this tack felt discourteous to the CMAs who felt overworked and financially and academically undervalued (not having received permanent contracts after 10 years of service, nor academic recognition in published papers). In the face of their expressed frustration, it felt discourteous to use ‘fun’ as an inducement.^5^ Besides, the members of the research team who might learn most from interacting with participants were more senior staff who encountered the community least in their everyday work. I sensed that ‘fun’ would not override the perceived importance of their work.

Nonetheless, ‘fun’ and ‘play’ were necessary characteristics of the spaces and interactions fostered within Sacred Water and Jeewan Jal. I observed, however, that what I expected to be a positive experience could induce anxiety for researchers; which I will now address.

Accompanying these pronounced hierarchies of professional status, interviews revealed that different researchers held different preferred ‘target groups’ and associated purposes for engagement. Their preference appeared to be aligned with their professional position. For the unit director, influencing medical practice and policy at a national and district level was the overriding interpretation (and concern):

Director: So our team should engage up and down the engagement spectrum. On the top would be the Ministry of Health. You know you have to aim big! You have to say ‘look, we’ve done this research and we have published in these journals…’ The lowest is going down to the districts.

To Dr B, engagement was very much about clinical relationships and the instrumental needs of research.

Dr B: The origin of the community engagement concept … is that, say in Africa if you were doing something like the typhoid burden study … in any mass community engagement you would have leaders and consent would be taken from community leaders and their consent would mean, ‘Okay you can do it in the community’ … We deal with individuals and not with communities…

Dr E was the staff member who gave most time to Jeewan Jal. I asked why she felt engagement was important. Her rationale concerned moral duty:

Dr E: It’s giving back to the community. The science, the results, something that you know and then making it aware [*sic*] in the community in a very different way not in a scientific way but in an easy way so that they could understand it easily.

Those who did not recognise their favoured target groups or justification for engagement within the opportunities offered were less likely to be involved. The director, who indicated an interest in engaging with policy, was verbally supportive, yet neither interacted with nor attended any project events. I suspect that he did not recognise these opportunities as being intended to inform him or other policy influencers, but that rather he understood the projects as community education, with little potential for broader reach. Dr B, who was keen to recruit research subjects, tended towards disseminating promotional information, whereas Dr E felt a moral responsibility to disseminate research knowledge within the community.

It is worth noting that in all instances presented above, the emphasis was on an engagement mode that favoured dissemination and education without incorporating an imperative for researchers to listen and understand, indicating a persisting ‘deficit’ attitude and disinterest in local knowledge, values and behaviours.

#### Comfort with creative and dialogic engagement formats

Helguera (2011, p. 45) tabulated two features of engaged encounters: openness or closedness of the format, and directedness of the subject. An open format is one in which participants are creative, in that they have a lot of input into a conversation, such as in a brainstorming activity. A closed format would include a lecture or a speech. I used a mixture of both open and closed formats throughout the project, depending on the project stage, topic and task. Open interactions brought research staff and community participants together without any obvious required outcome other than the very tangible and practical. Conversation was unpredictable, which, permitted some playful, creative and productive exchanges. Open-format encounters included the exhibition launch celebrations and trips to collect prop materials for the play from the hospital. Within Jeewan Jal, an open-format activity included a bus journey to Lele, a village half an hour from Patan and the place of a worshipped water source. Women’s group members, young adults and research staff sat together for the journey. Conversation topics were not directed, but the proximity of seating allowed for and encouraged interaction, with serendipitous results. For example, some community participants learned about, and subsequently attained data collection positions at, OUCRU-NP.

**Figure 1.**
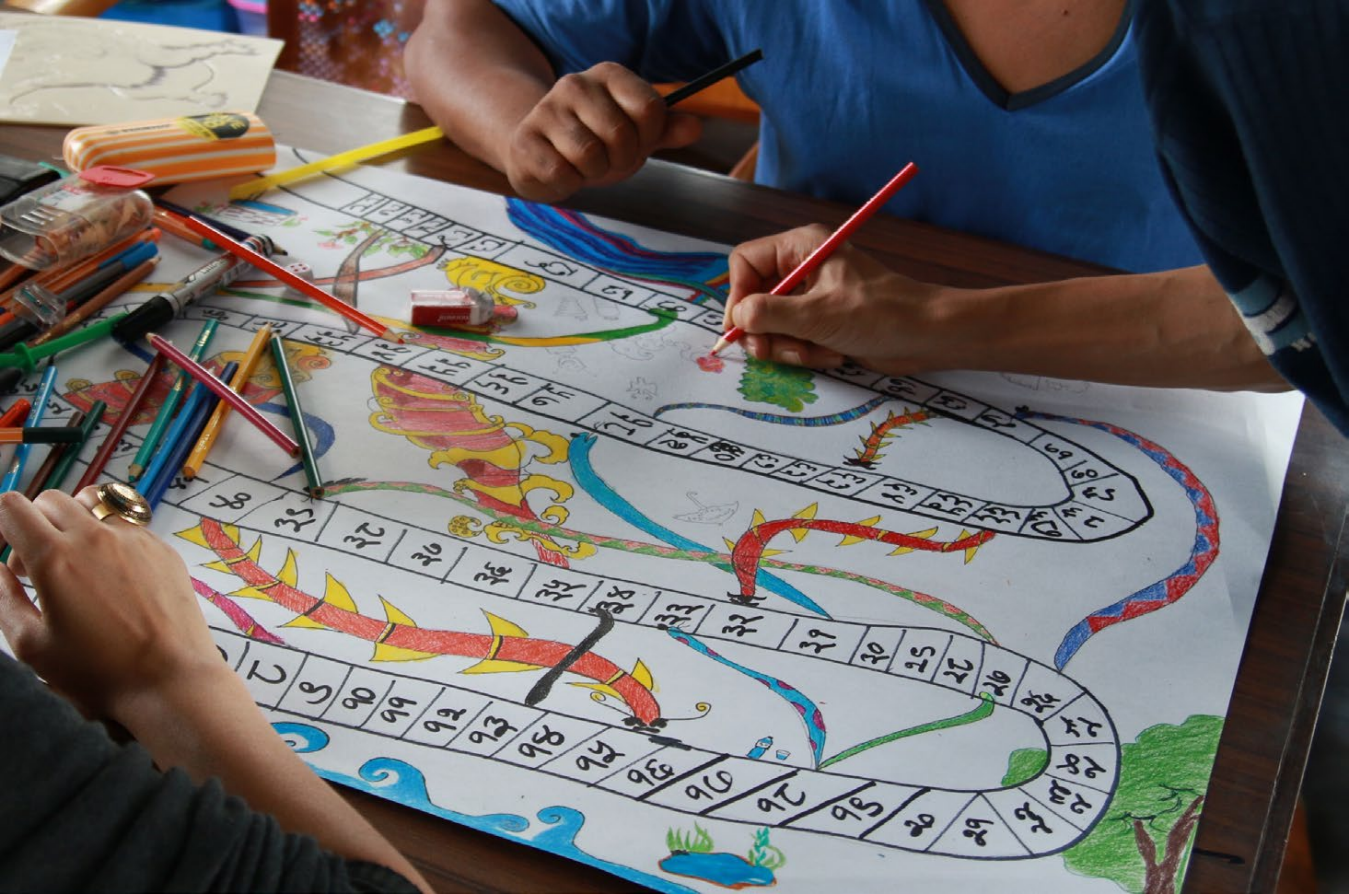
Project participants designing the Nags and Makars board game (based on Snakes and Ladders) (Aggett, 2015a).

At other times an open format was used, but with a more directed subject matter. For example, in Jeewan Jal, research staff were invited to donate water and an accompanying story to our ‘museum of water’ (a collection of donated water samples with accompanying stories illustrating water and how it features in daily life).^6^ They were also invited to offer ideas for reward and penalty cards to be used within our board game, entitled ‘Nags and Makars’ (see Figure 1) (an equivalent to the game ‘Snakes and Ladders’, seen played in Patan’s communal spaces). Such requests were given in a weekly team meeting and would be followed by an email and face-to-face prompting.

Within both projects, researcher participation was always voluntary. The lead artist would set up the engagement opportunity, invite research staff and hope that they would attend. Frequency and degree of researcher involvement was notably unpredictable. Whilst some team members participated occasionally, garnering longer term or iterative input from any one researcher was difficult. In Sacred Water, invitations to staff to participate in workshops with participants or separate workshops of their own met little positive response. During the Jeewan Jal project, I found that more open formats that invited creative input barely generated any researcher input – only three of the 20 staff donated water to the Museum of Water, and none contributed ideas to our board game. Of the various invitations into engaged interactions, it was the more closed formats, especially if focused on biomedical subject matter, which were the most conducive to researcher input.

This lack of input was perhaps because research staff were intimidated or confused by requests for creative thought. As Dr E reflected,

The water museum was quite new to me and I was quite impressed with that. Although I couldn’t give you the water because I didn’t know what I should … [*sic*] But I think that the water museum was a very good idea.

The lack of capacity or the insecurity of research staff preventing them from generating new ideas was explained to me as being a consequence of the Nepali education system, which employs rote learning and hampers the ability to question and think creatively:

Director: That’s the result of rote learning. So the result of rote learning is ah, suffocation. You know? You are stuck in your own rote learning ‘five causes of this ten causes of that’, when it could be something else.

Siân: And that impacted on people’s degree of comfort in doing something as open as I was trying to do?

Director: Exactly. Exactly! And if you encountered resistance in the UK you would encounter more resistance here because of how we have a packaged deal in learning.

This was perhaps compounded by a cultural avoidance of voicing questions, especially amongst women:

Dr R: In the Nepali culture you don’t question especially if you have a male doctor who has worked for 10 years.

Dr R hinted that the challenge I faced might also be due to epistemological differences between the arts and humanities and her epidemiological approach, which tended to look at causal links to proximal and predefined risk factors (Nichter, 2008).

Dr R: for me everything is facts and everything is in little boxes and then I have got a definite goal … I feel like with social sciences and arts it’s very open and you have to be open to get somewhere and I think it is that openness that makes me scared.

Finally, Dr R indicated that there was a conceptual barrier against participating in the arts for those who identify as scientists:

Dr R: … there is like this age-old thing … with [Standard Leaving Certificates], if you get really good marks and pass in the first division then you study science. If you are average, second division, you study business commerce and if you fail or are really at the bottom with your marks then you go into arts and social sciences. So here the doctors are like ‘Oh, we don’t get involved with arts, it’s not for us...’

Where moments of creativity were inspired in researchers, it was when there was no obvious pressure for creative performance. Such instances arose in situations where research staff were invited into closed-format interactions where they might expect to educate community members; however, careful facilitation permitted open musing with community participants within these. During such a situation, Dr E (the doctor who had not felt able to contribute water to the museum of water) showed creative ability. She came to talk about her work in the research lab with Jeewan Jal participants who wanted to present the storyline for their play. The group that day was small, and conversation was exploratory and relaxed when Dr E suggested we name a fictional antibiotic which featured in our story ‘*Wasa*-*cilin*’, marrying the standard antibiotic suffix with the Newari word for medicine.

Another process that included notable elements of exploration, creativity and two-way interaction built on a pedagogic structure used in medical school and so was familiar to medical researchers: a directed conversation with discussion points. The structure was open enough to enable conversation within the space, yet closed enough for me to facilitate. Dr R presented her water filter trial design, along with some unexplained questions and challenges she faced in her work. There was little requirement for medical researchers to step out of their area of expertise in this interaction. As part of the process, groups involving both community members and research staff were invited to have a dialogue and to think of explanations for phenomena such as seasonal typhoid patterns or why young men fell ill more than women. Dr R responded respectfully to the group’s hypotheses, such as that perhaps migrant males fell ill more readily because they were more likely to drink water outside of the home than women, noting within their responses values, relationships and practices that she had not previously known. In the interaction, she witnessed discriminatory attitudes towards migrant families and their unclean behaviours. This was something she had not learned about in four years of community-based work. She reflected on this, ‘That was horrible to hear, to be honest I had never heard that argument before. Even though I went in there and we were distributing water with the water tankers I never saw that’. Later in the session, Dr B arrived. He did not follow the suggested dialogic structure. Rather than discussing research findings and processes, he stood in front of the group and spoke of careers in research in a didactic style, perhaps replicating that in which he had been taught during medical training.

**Figure 2.**
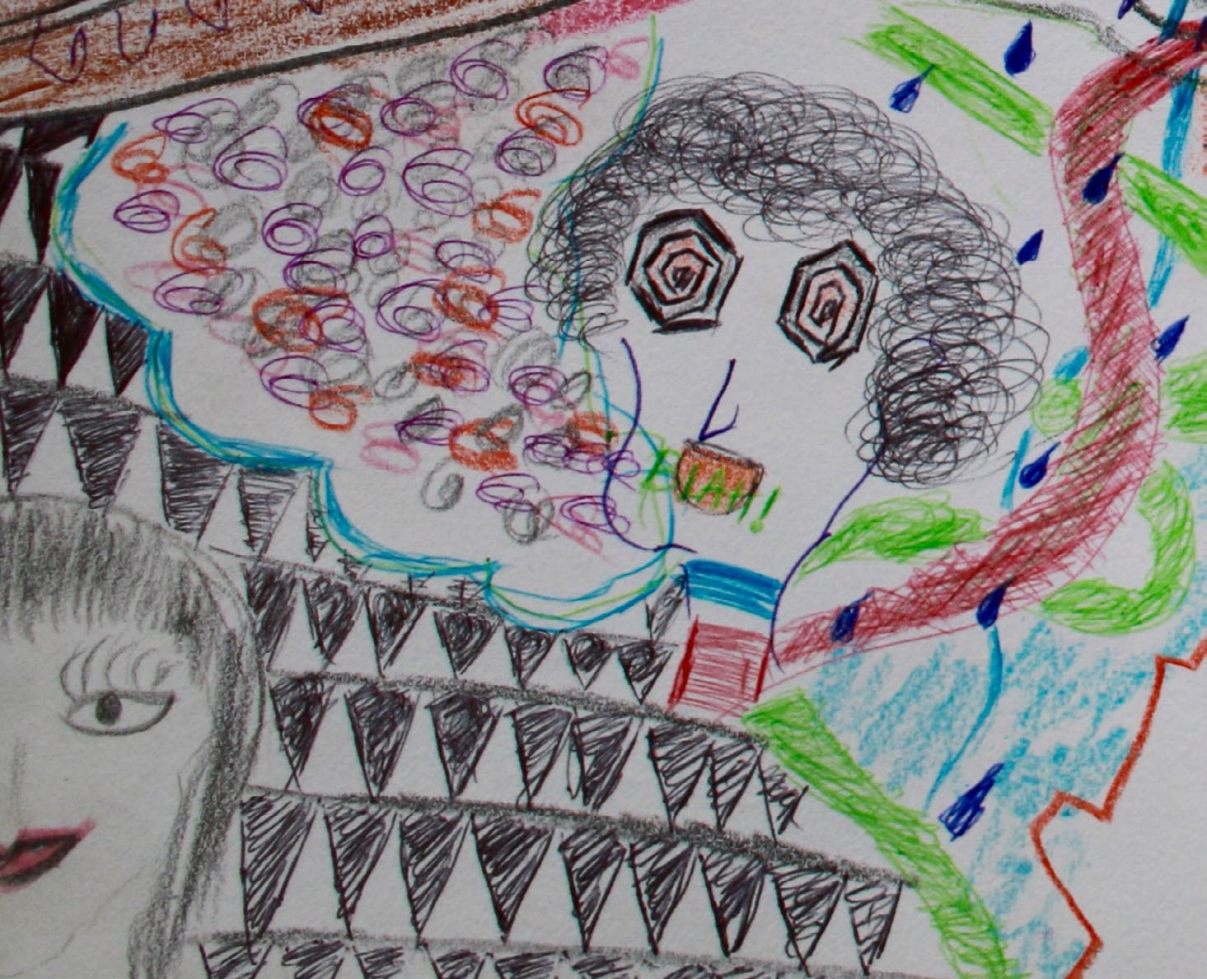
Representation of researcher engagement styles (Aggett, 2015b).

In a separate workshop, after this session, community participants depicted their experience of the discussion in drawing. One group’s illustration clearly represented their experiences of the different approaches:

The illustrator explained the picture (see Figure 2), saying:

The two faces at the corner symbolise the two scientists we interacted with at Patan Hospital. The lady figure at the bottom is Dr R. The white and black background around her represents her study. She shared all her results and outcomes [these are illustrated in white], but she did not have answers to some questions. She was really honest and clear about it [this is illustrated in black]. Dr B on the other hand discussed research methods. As shown in the doodle it was a little confusing for me. (Niraula, 2016)

In sum, closed formats, which replicated educational scenarios with which research staff were familiar (facilitated to retain a degree of openness) appeared to create less anxiety in researchers than open formats (especially when the conversation topic moved outside of their perceived area of expertise). By contrast, facilitating open engagements was easier with community participants, who met more regularly. Therefore, perhaps counter-intuitively, ‘semi-closed’ formats were most successful in setting up exploratory exchange. Moreover, research staff were better at contributing creatively in informal situations in which they felt unexamined. Didactic roles may have been more comfortable for some of the researchers, but these were less successful in creating two-way exchanges and in conveying information, perhaps because the content was not grounded in participants’ existing knowledge or interests but rather in assumptions of these.

## Conclusion

Sacred Water and Jeewan Jal were carried out in response to challenges faced by biomedical researchers in understanding responses to proposed research within Patan. It was hoped that the engagement projects would enable researchers to deepen their understanding of the subjectivities of people within Patan, encouraging more critical thought and perhaps even restructuring how researchers framed research problems and challenges within research design. Neither project, however, succeeded in garnering the degree of involvement from research teams that was required for such self-reflection. Researchers tended to see community resistance as something correctable through education, rather than through engaging in two-way communication (Wynne, 2014).

To some degree, the challenges described in this paper are inherent to any process that brings groups together across power differentials, interests and ways of understanding the world (Helguera, 2011; Rooke, 2013). However, I also suggest that they are particular to engagement in biomedical research contexts, especially in low-income settings where deep hierarchies prevail.

Researchers, perhaps without even realising it, conducted cost–benefit analyses of engagement processes and – for the most part – found that inducement to engage was lacking. In the projects described, both the funders and the researchers themselves indicated that they wanted engagement, but institutional, funder and disciplinary reward systems discouraged involvement. There was no accountability for involvement (or non-involvement), nor were there obvious career-benefitting reasons to be involved. Messaging around funding institutions’ policies and priorities, whilst not having complete power over researchers’ attitudes towards engagement, can be influential (Palmer & Schibeci, 2012). Involvement in engagement processes was further discouraged by institutional hierarchies, such as the sense that the arts were for people who were less academically gifted than scientists, compounded by an insecurity and avoidance of approaches and formats deemed appropriate for meaningful engagement.

When medical researchers did engage, dialogical interactions were often undermined by a ‘deficit’ attitude, in which the purpose of engagement was assumed to be about educating people about research findings – accompanied by the assumption that the public would view research more favourably if they were better informed. If, as a thought experiment, we flip this model over, the corresponding argument would be that researcher involvement in engagement processes only requires that the researchers are sufficiently educated to understand the benefits of their involvement. This paper has shown that this is not the case; there are multiple factors that play into researcher resistance to involvement in engagement, and whilst education as to the value of engagement might be one factor, it is not a deciding one.

Efforts at generating engagement were taxing. As a facilitator, I found myself supporting research staff to overcome their discomfort with processes that did not have defined outcomes, needing to curate an unexpected variety of open and closed interactions in order to generate interaction, and having to find subtle mechanisms to engage researchers in dialogue and two-way communications. These were demanding and unanticipated tasks, which were not accounted for in project plans, nor resource and time allocation. These tasks are easily overlooked if we only emphasise the ‘community’ of ‘community engagement with research’.

To conclude, this paper is intended to advance debate in the field of community engagement with global health research by addressing a much-overlooked dimension (the engagement of researchers themselves). Despite the challenges described above, I maintain that the critical approach taken by a strong participatory arts practitioner can allow for in-practice resistance to the expectation of one-way didactic engagement approaches by creating spaces for dialogue between actors who view things from differing vantage points. In order for this to happen, a number of conditions are required. There needs to be a recognition – from funders, researchers and engagement practitioners alike – that the work required of the facilitator to achieve engagement is highly demanding and unpredictable. Funding institutions and other associated academic institutions need to be aware of the role they play in encouraging engagement, and could even consider playing more of a supportive curatorial/brokering role between practitioners and institutions. Medical researchers need better awareness of the various guiding principles, aims and objectives for engagement, lest activities be destined to slip towards a deficit mode, which most engagement practitioners are keen to leave in the past (Wynne, 2014). Clearer communication and opportunities for experiential and accredited researcher training may support this. Finally, researcher involvement in ‘meaningful engagement’ over tokenistic engagement efforts must be recognised and rewarded where it takes place.

In order to develop more pointed strategies, we need an equitable, inclusive and critical conversation between funders, research institutions and engagement practitioners about what fairly rewarded and meaningful engagement might look like, and the structural changes required to support these. This ought to include conversations around the possibilities, challenges and responsibilities of involving practitioners from outside of medical research institutions, such as participatory artists/facilitators. Without this, the potentials of independent practitioner-led engagement maybe destined to fail, as they are misunderstood and easily slip to the peripheries of the knowledge project, with it being impossible to encourage anything more than shallow and tokenistic involvement from researchers, thus proliferating rather than contesting existing power structures.

## Disclosure statement

No potential conflict of interest was reported by the author.

## Funding

This work was supported by The Wellcome Trust.

## Notes

1. http://www.sacredwaternepal.com

2. https://www.jeewanjal.com/about-the-project

3. Apart from a few exceptions, such as MIRA’s (Mother and Infant Research Association) participatory film with rural women’s groups (See https://vimeo.com/33243824) and the Nick Simmons Institute’s radio docudrama series of health workers’ stories (see http://www.nsi.edu.np/?option=com_content&func=details&id=8#.WQr_NlPys1 g).

4. I had a long-standing relationship with OUCRU-VN and OUCRU-NP, having worked with both of these programmes at earlier points in my career.

5. Advice from the senior members of the OUCRU team was that we ought not to pay staff members, as engagement was considered part of their professional responsibility.

6. The Patan Museum of water was inspired by Amy Sharrock’s Museum of water (http://www.museumofwater.co.uk).
